# Analytical Validation of NavDx+Gyn, a cfDNA-Based Fragmentomic Profiling Assay for HPV-Driven Gynecologic Cancers

**DOI:** 10.3390/diagnostics15070825

**Published:** 2025-03-25

**Authors:** Joshua Hutcheson, David Conway, Sunil Kumar, Chloe Wiseman, Syandan Chakraborty, Evgeny Skrypkin, Michael Horan, Alicia Gunning, Cassin Kimmel Williams, Charlotte Kuperwasser, Stephen P. Naber, Piyush B. Gupta

**Affiliations:** Naveris, Inc., Waltham, MA 02451, USA

**Keywords:** NavDx+Gyn, cervical cancer, vaginal cancer, vulvar cancer, HPV, papillomavirus

## Abstract

**Background/Objectives**: The NavDx+Gyn blood test detects and quantifies fourteen HPV types in various sample types to provide a reliable means of detecting and monitoring HPV-driven gynecologic cancers. NavDx+Gyn is an extension of the NavDx assay, which identifies five high-risk HPV types. NavDx has been clinically validated in multiple independent studies for the surveillance of HPV-driven oropharyngeal cancer and has been integrated into clinical practice by over 1300 healthcare providers at over 500 medical sites in the US. The NavDx+Gyn assay incorporates an analysis of nine additional high-risk HPV types. Here, we report a detailed analytical validation of the NavDx+Gyn assay for use in cervical, vaginal, and vulvar cancer patients to detect fourteen high-risk HPV types related to HPV-driven gynecologic cancers. **Methods**: Parameters include specificity as measured by limits of blank (LoBs) and sensitivity illustrated via limits of detection and quantitation (LoDs and LoQs). **Results**: The LoBs were between 0 and 0.0926 copies/μL, LoDs were 0.1009 to 0.3147 copies/μL, and LoQs were 0.1009 to 0.3147 copies/μL, demonstrating the high analytic sensitivity and specificity provided by NavDx+Gyn. In-depth evaluations, including accuracy and intra- and inter-assay precision studies, were shown to be within acceptable ranges. Regression analysis revealed a high degree of correlation between expected and effective concentrations, demonstrating excellent linearity (R^2^ > 0.99) across a broad range of analyte concentrations. **Conclusions**: These results demonstrate that NavDx+Gyn accurately and reproducibly detects fourteen types of high-risk HPV, which aids in the diagnosis and surveillance of the vast majority of HPV-driven gynecologic cancers.

## 1. Introduction

Human papillomavirus (HPV) is the causative agent for most squamous anogenital and oropharyngeal cancers [1]. HPV-driven carcinomas, including cervical, vaginal, and vulvar cancers, arise from alterations in the cervical squamous epithelial or endocervical mucosal cells due to chronic HPV infection [1,2,3,4,5]. In 2021, the latest year with available data, an estimated 19,345 new cases of cancer of the cervix, vagina, and vulva were reported in the United States. This included 12,536 cases of cervical cancer, 1372 cases of vaginal cancer, and 5860 cases of vulvar cancer [6]. Of these cancers, 16,345 cases (10% of cervical cancers and 70% of vaginal and vulvar cancers) are estimated to be associated with HPV, corresponding to an incidence rate of approximately 9 cases per 100,000 women [7]. The overall incidence rate of cancer involving the cervix, vagina, and vulva, including non-HPV-related cases, was 10.7 per 100,000 women, with a breakdown of 7.4 for cervical cancer, 0.6 for vaginal cancer, and 2.7 for vulvar cancer per 100,000 women [6]. While vaccination and screening efforts in many high-income countries have reduced the incidences of these cancers over the past twenty years, lower- and middle-income countries continue to face high incidence and mortality rates [8,9,10].

The recurrence rate in patients with early-stage cervical cancer is 10–15%, while the recurrence for more advanced-staged cervical carcinomas can be as high as 70% [11]. Early detection of residual and recurrent HPV-associated cervical, vaginal, and vulvar cancers facilitates the timely initiation of adjunctive therapy, thereby supporting improved clinical outcomes. For patients with HPV-associated gynecologic cancers, the recommended standard of care is stage-dependent and includes surgery and/or various modalities of chemotherapy and radiotherapy [12]. Post-therapy surveillance to assess treatment response utilizes interval clinical examination, cytology screening, and stage-dependent imaging, which may be based on fertility status. The complexity and variability of current surveillance protocols underscore the need for a reliable surveillance test that indicates recurrent HPV-driven gynecologic cancer and is easily accessible to patients and clinicians. A plasma-based test that identifies circulating HPV tumor DNA (“liquid biopsy”) to detect gynecologic cancer recurrence could meet this need.

Circulating tumor DNA (ctDNA) released by dying cancer cells represents a source of cervical cancer tumor genomic biomarkers that are accessible by routine phlebotomy [13,14]. Liquid biopsy assays that detect circulating ctDNA are an increasingly common tool that provides genomic profiling results for assessing tumor status and directing therapy [15,16,17]. For patients with HPV-associated tumors, the transcription products of HPV viral genes E6 and E7 are primary drivers of malignancy [18,19,20,21,22,23]. A blood-based assay that detects circulating fragments of HPV tumor DNA could provide a less invasive, more sensitive, and specific method to detect the presence of residual or recurrent tumors [24,25,26,27,28,29,30,31,32,33,34,35,36]. We have, therefore, developed NavDx+Gyn, a blood-based molecular assay to detect circulating HPV DNA arising from HPV-driven gynecologic carcinomas. NavDx+Gyn is compatible with most sample types, including but not limited to blood, plasma, serum, FFPE tissue, saliva, and cervical swabs. NavDx+Gyn detects fourteen HPV types commonly associated with gynecologic carcinomas and is an extension of the previous analytically and clinically validated NavDx test, which detects five high-risk HPV types [37,38,39,40,41,42,43,44]. Oropharyngeal cancer is mainly associated with the five high-risk HPV strains covered by the NavDx test. The new NavDx+Gyn test now includes nine additional HPV subtypes, enhancing its utility in diagnosing other HPV-related cancers, including cervical, vaginal, and vulvar cancers, where these additional strains are commonly found.

Here, we detail the analytical validation of the NavDx+Gyn assay adhering to the standards set by the Clinical Laboratory Improvement Amendments (CLIA 1988) for laboratory-developed tests and the corresponding guidelines of the Clinical and Laboratory Standards Institute. The evaluation included the limit of the blank (LoB), limit of quantification (LoQ), limit of detection (LoD), intra-assay and inter-assay precision, accuracy, and assay linearity.

## 2. Materials and Methods

### 2.1. Test Characteristics

NavDx+Gyn is a high-complexity laboratory-developed test that helps in the detection of gynecologic cancer driven by human papillomavirus (HPV). NavDx+Gyn is a droplet digital PCR-based assay that can utilize DNA sourced from multiple sample types, including plasma, FFPE tissue, saliva, and cervical and anal swabs. Using 34 DNA biomarkers, NavDx+Gyn detects and profiles the fragmentation pattern of HPV DNA through droplet digital PCR (ddPCR). A quantitative algorithm calculates an HPV DNA Score by differentially weighing HPV DNA fragments according to size, reflecting the total amount of HPV DNA. NavDx+Gyn detects and identifies fourteen HPV types commonly associated with gynecologic cancer: 16, 18, 31, 33, 35, 39, 45, 51, 52, 56, 58, 59, 66, and 68. The detection of an internal control gene, ESR1, is used for quality control on each specimen.

### 2.2. Bioinformatics

The NavDx+Gyn assay analyzes the isolated DNA by ddPCR using 34 probes and 68 primers targeting genomic regions in fourteen high-risk HPV strains. Another probe with two primers directed at *ESR1* served as the sample’s quality control. Each probe–primer pair represents a specific amplicon target within the HPV genome, and the collective set of probes and primers is designed to support the detection and quantification of fourteen HPV types by computational analysis. The HPV DNA Score was calculated using the method previously described [44].

### 2.3. Validation Materials

Synthetic HPV DNA (Integrated DNA Technologies, Coralville, IA, USA) encoding for the target-specific DNA regions of HPV types 16, 18, 31, 33, 35, 39, 45, 51, 52, 56, 58, 59, 66, and 68 in TE buffer or water with polyA was used as a reference standard for validating the assay. The expected reference standard concentrations for all HPV types used in the validation study are detailed in Appendix A. Briefly, for accuracy, precision, and linearity, concentrations ranged from ~4000 to 4 copies/µL. These dilutions were prepared and measured by ddPCR to provide the HPV DNA Scores found in the top half of Appendix A. For validation of sensitivity, a series of lower-concentration dilutions were prepared by diluting a parent stock whose concentration was validated by ddPCR. For HPV16, the dilution series HPV DNA Scores ranged from 20 to 0.04, while all other HPV types were from 20 to 0.17 copies/µL. The reference standards used for LoQ and LoD determinations are the expected dilutions expressed in copies/µL.

A total of 42 no-template controls (NTCs) were run alongside positive controls to determine the limits of blank. A dedicated technologist (Technologist “A”) ran the analytical levels in triplicate across three non-consecutive days to establish accuracy, method precision, and linearity. Technologist “B” ran the series once on a different day to perform an additional run of the analytical levels to determine intermediate precision. All ddPCRs were performed on the Bio-Rad QX200 AutoDG ddPCR system with the software version 1.7.4.0917 (Bio-Rad, Hercules, CA, USA).

### 2.4. Determination of Assay Performance Characteristics

#### 2.4.1. Specificity (Limit of Blank)

The specificity of the NavDx+Gyn assay was determined by calculating the limit of blank (LoB) following the Clinical and Laboratory Standards Institute (CLSI) guidelines, adapted for ddPCR. The LoB is defined as the maximum quantity expected to be observed for a blank sample (zero analyte copies) with a stated probability. No-template controls (NTC), consisting of molecular-grade water devoid of DNA, were analyzed to determine specificity. A total of 42 NTC samples were tested to determine the LoB for each HPV DNA analyte.

The limit of blank was calculated as follows: LoB = meanNTC + 1.645(SD_NTC_).

#### 2.4.2. Sensitivity (Detection Limit)

The limit of detection (LoD) was determined by using sequence-verified synthetic HPV Target Sequence DNA to measure the absolute quantification of HPV DNA of types 16, 18, 31, 33, 35, 39, 45, 51, 52, 56, 58, 59, 66, and 68 via digital droplet PCR (ddPCR). To determine the limit of quantification for each assay, target sequence solutions for each analyte were prepared, and three replicates of twelve titration standards were used (see Appendix A for full details of concentrations used). The limit of quantitation (LoQ) is the lowest concentration at which the analyte can be reliably detected, as determined by acceptable %CVs [45]. The LoQ and LoD were defined following the CLSI guidelines adapted for ddPCR. The LoD was calculated using the following formula from reference [46]: LoD = LoB + 1.645(SD_Low concentration sample_).

#### 2.4.3. Accuracy, Precision, and Linearity

A series of dilutions of synthetic HPV DNA were used to determine analytical accuracy, method precision, intermediate precision, and linearity for each HPV type. The samples were prepared by diluting the HPV target sequence to specified concentrations. Final concentrations/HPV DNA Scores ranged between ~4000 copies/µL and 4 copies/µL for all HPV types (see Appendix A for details on specific concentrations/HPV DNA Scores used for each HPV type). To assess analytical accuracy, analyte concentration was measured in replicates on three distinct days using samples with known analyte levels, with results reported as the percent recovery of the known concentration. Intra-assay precision (method precision or repeatability) was determined by measuring variation observed by a single technologist over three non-sequential days. Inter-assay (or intermediate) precision refers to variations within the lab, including differences in days, technologists, and instrumentation. Each of the control sample solutions was tested in triplicate at five (for HPV16) or seven (for non-HPV16 type) different concentrations/HPV DNA Scores on three different days to determine intra-assay precision. Inter-assay precision was assessed by testing on an additional day with a different technologist. The analysis of samples included calculating the coefficients of variation (% CV) for the mean effective concentrations in both studies. Linearity assesses the concentration range over which the response of a calculated measurement is directly proportional to the analyte concentration, typically evaluated through regression analysis, with a good R^2^ value indicating a strong linear correlation between concentration and response. The linearity of NavDx+Gyn was measured by preparing five (HPV-16) or seven (non-HPV-16 type) standard solutions of analyte for each type as described in the methods for accuracy and method precision. Samples were analyzed, and linear regression analysis was carried out to examine the correlation between analyte concentration and the corresponding signal response (or effective concentration).

## 3. Results

### 3.1. Detection Capability

#### 3.1.1. Specificity (Limit of Blank)

A total of 42 no-template control (NTC) replicates were analyzed to determine the limit of blank (LoB) for the HPV assays. The majority of non-template control (NTC) replicates produced negative results, with a limited number exhibiting low-level values. Based on the observed data, the calculated LoB values for the fourteen assays for HPV types 16, 18, 31, 33, 35, 39, 45, 51, 52, 56, 58, 59, 66, and 68 are summarized in Table 1.

#### 3.1.2. Sensitivity (Detection Limit) and Limit of Quantitation

The limit of quantitation (LoQ) was determined for each HPV type using titration samples from 20 to 0.17 (for HPV types 18, 31, 33, 35, 39, 45, 51, 52, 56, 58, 59, 66, and 68) or 20 to 0.04 copies/μL (for HPV-16) (see Appendix A for details on concentrations used). The limit of detection (LoD) for each variant was determined based on the calculated limit of blank (LoB) and the standard deviation of a low-concentration sample, ensuring a minimum detection rate of 70% across replicates. LoDs for the fourteen HPV types tested ranged from 0.1009 to 0.3147 copies/μL. The LoQ for HPV-16 was measured as 0.1009 copies/μL and ranged from 0.1175 to 0.3147 copies/μL for all other HPV types tested. The limits of detection (LoDs) and limits of quantitation (LoQs) for detecting fourteen HPV types are summarized in Table 1.

### 3.2. Analytical Accuracy

Standard control samples of the prepared HPV target sequence for all HPV types (HPV types 16, 18, 31, 33, 35, 39, 45, 51, 52, 56, 58, 59, 66, and 68) were evaluated at five or seven concentrations, ranging from approximately 4000 to 4 copies per μL (refer to Appendix A for detailed concentration information). The expected recovery was within ±20% of the target concentration, with mean percentage recoveries for all tested HPV types ranging from 83.4% to 113.1%. The only exception was an outlier of 125.3%, observed at the lowest concentration for HPV-18. Table 2 summarizes the mean percent recoveries for each HPV type, demonstrating analytical accuracy.

### 3.3. Precision Studies

Intra-assay precision, or method precision, was evaluated by measuring the effective concentrations of five or seven dilutions for each HPV type over three days. The coefficient of variations (%CV) for all HPV types were below 20%, ranging from 0.3% to 18.2%. Detailed results for method precision at varying concentrations for each HPV type are presented in Table 3.

Inter-assay or intermediate precision was evaluated by assessing variation within the laboratory across different technologists and days. The results obtained over multiple days were compared statistically. The coefficient of variation (%CV) for all HPV types remained below 20% across all concentration levels, ranging from 1.0% to 16.7%. Detailed results from the intermediate precision studies are presented in Table 4.

### 3.4. Linearity

**Figure 1:**
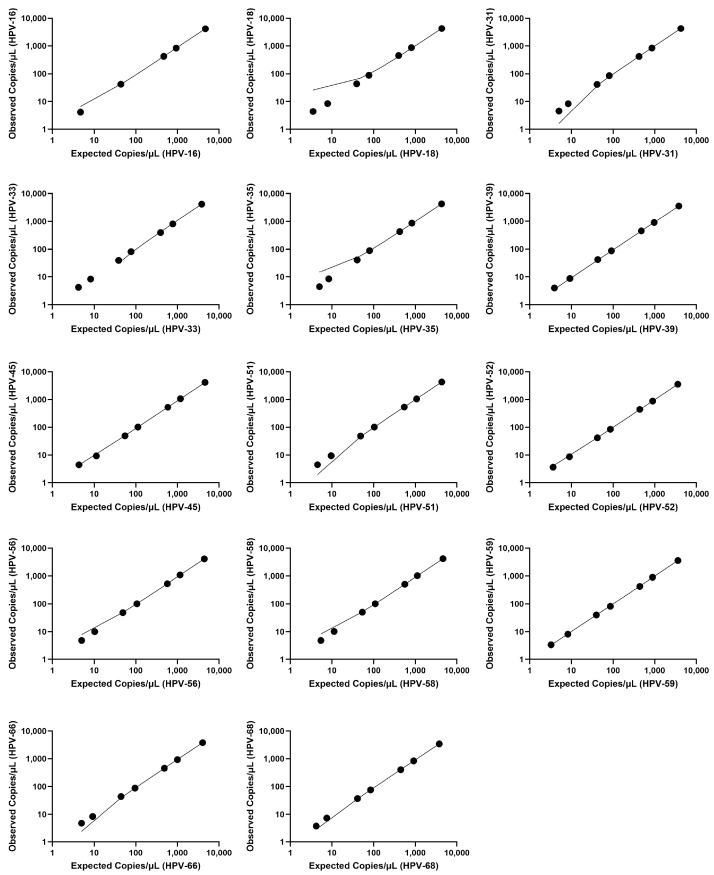
Linearity plots of mean measurements obtained over three days for HPV types 16, 18, 31, 33, 35, 39, 45, 51, 52, 56, 58, 59, 66, and 68 at five or seven concentrations. The corresponding equations and R^2^ values are provided in Table 5.

The linearity of the NavDx+Gyn test was evaluated to determine its ability to produce responses proportional to the analyte standard concentration. The slopes observed for expected versus effective concentrations spanned from 0.8868 to 1.088, with intercepts spanning from −10.92 to 22.50. Coefficients of determination (R^2^) exceeded 0.99 for all HPV type regression plots. Linear plots representing the mean concentrations across three days are shown in Figure 1. Table 5 provides the equations and corresponding R^2^ values for each HPV type. Evidence of linearity (R^2^ > 0.99) is further demonstrated with the LoQ results, extending to the lowest range tested (down to less than 1 copy/µL; refer to Appendix B).

## 4. Discussion

Human papillomavirus (HPV)-driven gynecologic carcinoma is continuing to increase globally in incidence and morbidity, even while vaccination and treatments improve among developed nations [8,9]. Reliable and accessible testing with minimal inconvenience and expense for patients and clinical practice sites is indispensable for early tumor recurrence detection and the continuous monitoring of HPV-driven gynecologic cancers. NavDx+Gyn is an extension of NavDx, a proprietary laboratory-developed diagnostic assay designed and validated to detect HPV DNA originating in oropharyngeal carcinoma, referred to as circulating tumor tissue modified viral (TTMV)-HPV DNA [37,38,39,40,41,42,43,44]. NavDx+Gyn is designed for monitoring patients with HPV-driven cervical, vulvar, and vaginal cancers for minimal residual, recurrent, and distant metastatic gynecologic cancer. It detects and quantifies circulating HPV DNA from fourteen high-risk HPV types known to be associated with cervical, vaginal, and vulvar cancer [47,48,49,50,51]. Providing an accessible and reliable blood test with a low impact on patients and clinical sites, the NavDx+Gyn assay can detect HPV-driven gynecologic cancer recurrence much earlier than current clinical surveillance practices.

Liquid biopsy assays for cancer detection that analyze cell-free DNA from blood specimens face several challenges, including low levels of tumor-derived DNA, a short half-life in circulation, and non-tumor sources of cell-free DNA. In addition to interference from cfDNA derived from white blood cells and other non-tumor sources, technical variability in sample processing, extraction, and analytic methods can affect results. Biological conditions including inflammation, infection, or pregnancy can also affect the quantitative relationship between tumor burden and circulating tumor-derived cell-free DNA. Furthermore, pre-analytical factors such as sample collection, storage, and handling are crucial for maintaining cell-free DNA integrity. However, advancements in liquid biopsy technologies have significantly improved the detection and analysis of tumor-derived cell-free DNA. Digital PCR, for instance, is a highly sensitive method capable of detecting even single copies of target DNA, addressing issues of low abundance when combined with multiple unique biomarkers and methods to assess fragment size. Enhanced techniques for sample collection, preservation, and extraction have also been critical in preserving the quality and quantity of cell-free DNA. Additionally, the use of bioinformatics, statistics, and machine learning algorithms has improved the ability to differentiate tumor-derived cell-free DNA signals from background noise, thereby increasing specificity. These and other innovations continue to refine assays such as NavDx and NavDx+Gyn, enhancing their reliability and applicability in a clinical setting.

The specificity and detection limits of NavDx+Gyn for HPV DNA were assessed by measuring the limits of blank (LoBs), limits of quantitation (LoQs), and limits of detection (LoDs) for each relevant HPV type. The assay demonstrated excellent specificity, as indicated by LoBs ranging from 0 and 0.0926 copies/μL for the fourteen HPV types tested. The LoQs were measured with dilutions ranging from 20 to 0.04 copies/μL for HPV-16 and from 20 to 0.17 copies/μL for HPV types 18, 31, 33, 35, 39, 45, 51, 52, 56, 58, 59, 66, and 68, and were found to range between 0.1009 and 0.3147 copies/μL. The LoDs were derived from these data and established as 0.1009 to 0.3147 copies/μL for the tested HPV types, illustrating the high sensitivity of the NavDx+Gyn assay (Table 1).

A series of five or seven dilutions of HPV target sequences for all fourteen types (HPV types 16, 18, 31, 33, 35, 39, 45, 51, 52, 56, 58, 59, 66, and 68) were prepared to determine analytical accuracy (Table 2), method and intermediate precision (Table 3 and Table 4), and linearity (Figure 1). Analytical accuracy was within the specified ranges, and precision studies demonstrated acceptable within-run precision and intermediate precision. Regression analyses showed strict linearity for all tested HPV types, with R^2^ values greater than 0.99 (Table 5). Linearity was demonstrated up to the limit of quantitation (see Appendix B for details). In these cases, dilutions of specimens demonstrated a good correlation with the expected levels of the original diluted analyte throughout the full range. NavDx+Gyn was shown to produce results that are proportional to the analyte concentration across a wide concentration range. While reporting on the analytical validation of the NavDx+Gyn assay, this study does not examine this test’s clinical utility for early diagnosis, treatment efficacy, recurrence, intervention, or treatment changes. To assess clinical utility, further research will be necessary to evaluate the assay’s performance across various patient groups and clinical environments. Current and forthcoming clinical trials are designed to address these inquiries, aiming to provide crucial data concerning sensitivity, specificity, predictive values, and broader clinical impact.

These results provide analytical validation for NavDx+Gyn, demonstrating its analytic reliability as a laboratory-based assay for use in detecting and quantifying fourteen HPV types associated with cervical, vaginal, and vulvar cancers.

## 5. Patents

Gupta, G; Chera, BS; Kumar, S: Method for Quantifying DNA Fragments in a Sample by Size. US 11168373B2, 2021.

Gupta, G; Chera, BS; Kumar, S: Compositions and Methods for the Selective Detection of Tumor-Derived Viral DNA. US 11254989B2, 2022.

Gupta, G; Chera, BS; Kumar, S: Methods of identifying patients as having an increased likelihood of having a human papillomavirus (HPV)-associated cancer or recurrence of an HPV-associated cancer. US11788157B2, 2023.

## Figures and Tables

**Table 1:** Limit of blank (LoB), limit of detection (LoD), and limit of quantitation (LoQ) for each of the fourteen HPV types detected and quantified by the NavDx+Gyn assay, expressed in copies per microliter (copies/μL).

HPV Type	HPV DNA Score (Copies/μL)		
	LoB	LoD	LoQ
HPV-16	0.0134	0.1009	0.1009
HPV-18	0.0273	0.2552	0.2552
HPV-31	0.0621	0.2423	0.2423
HPV-33	0	0.2963	0.2963
HPV-35	0	0.3022	0.3040
HPV-39	0.0779	0.2726	0.2726
HPV-45	0.0282	0.2520	0.2520
HPV-51	0	0.1983	0.2699
HPV-52	0.0581	0.2489	0.2489
HPV-56	0	0.1255	0.1255
HPV-58	0.0926	0.3100	0.3100
HPV-59	0.0315	0.1848	0.1848
HPV-66	0.0612	0.3147	0.3147
HPV-68	0	0.2874	0.2874

**Table 2:** Analytical accuracy, expressed as mean percent recoveries for HPV types across a range of dilutions, calculated as the average over three days.

Conc. Level ^a^	Accuracy—Mean % Recovery ^b^													
	HPV Type													
	16	18	31	33	35	39	45	51	52	56	58	59	66	68
1	88.7	98.7	100.3	108.7	99.0	93.7	89.8	100.5	99.2	93.1	90.3	99.7	93.8	90.8
2	88.8	107.0	97.7	104.3	104.3	92.8	90.3	100.8	99.1	94.5	91.2	101.8	92.0	91.0
3	90.0	113.1	99.0	101.1	101.4	94.3	89.4	101.9	100.3	93.5	91.6	97.5	93.7	89.5
4	n/a	114.7	105.6	105.4	112.0	94.9	90.8	100.2	98.4	94.6	92.8	94.9	92.3	89.3
5	96.2	111.1	100.8	101.6	102.8	97.7	89.3	101.3	99.3	98.7	94.1	100.5	98.2	90.5
6	n/a	107.8	100.3	102.5	101.2	94.7	83.4	96.4	97.1	97.4	90.4	99.0	91.6	98.5
7	87.6	125.3	91.4	102.2	89.4	103.1	103.1	101.0	100.1	97.2	89.2	105.7	96.6	89.9

^a^ Refer to the concentrations listed in Appendix A. ^b^ Measurements were conducted in triplicate over three days by Technologist A.

**Table 3:** Intra-assay precision (repeatability) expressed as the coefficient of variation (%CV) of the averaged effective concentrations for the fourteen HPV types across multiple concentrations over three days.

Conc. Level ^a^	Intra-Assay/Method Precision—%CVs of Mean Effective Concentration (copies/μL) ^b^													
	HPV Type													
	16	18	31	33	35	39	45	51	52	56	58	59	66	68
1	1.1	5.5	2.5	2.4	1.6	1.6	4.2	1.6	2.8	4.5	0.9	2.0	4.9	2.1
2	1.2	1.9	2.2	4.7	1.6	3.3	3.9	2.1	1.0	4.9	2.7	3.3	2.3	2.4
3	1.2	4.2	4.9	3.6	3.7	1.2	5.2	3.2	4.7	2.5	5.0	2.9	3.4	2.9
4	n/a	4.0	4.0	3.5	2.3	2.2	7.6	1.3	3.1	6.8	7.3	4.8	2.3	1.9
5	1.4	6.2	1.5	3.3	5.2	2.8	4.0	3.8	3.5	5.0	6.0	2.7	5.5	5.0
6	n/a	5.3	4.4	7.6	7.8	1.3	10.9	6.9	6.1	7.2	8.3	1.2	13.8	6.8
7	3.9	7.4	2.7	18.2	9.4	13.7	13.5	3.0	7.5	12.5	12.5	3.8	3.7	0.3

^a^ Refer to the concentrations listed in Appendix A. ^b^ Measurements were conducted in triplicate over three days by Technologist A.

**Table 4:** Inter-assay (intermediate) precision (within-lab variation) expressed as the coefficient of variation (%CV) of the averaged effective concentrations for fourteen HPV types across multiple concentrations over four days.

Conc. Level ^a^	Inter-Assay/Intermediate Precision—%CVs of Mean Effective Concentration (copies/μL) ^b^													
	HPV Type													
	16	18	31	33	35	39	45	51	52	56	58	59	66	68
1	1.1	4.7	2.4	3.7	1.4	1.9	3.7	1.4	2.5	3.8	1.0	2.1	4.0	2.4
2	1.6	1.8	3.4	3.8	1.3	2.8	3.2	2.6	1.2	4.1	2.2	3.1	1.9	2.0
3	1.9	6.9	4.4	5.5	3.4	1.0	4.3	4.4	3.9	2.0	4.2	2.5	2.9	2.5
4	n/a	6.4	3.3	6.0	2.0	2.0	6.8	3.9	3.1	6.0	6.8	4.1	2.1	2.3
5	1.7	5.6	2.6	2.8	5.5	2.9	5.1	3.1	3.4	5.3	5.5	4.4	5.7	4.4
6	n/a	7.2	3.9	8.1	6.5	5.8	13.5	5.7	5.3	6.4	8.4	9.5	11.6	5.8
7	4.2	11.6	6.6	16.7	7.9	11.3	11.0	2.5	8.4	10.2	10.2	6.5	3.9	3.4

^a^ Refer to the concentrations listed in Appendix A. ^b^ Measurements were conducted in triplicate over three days by Technologist A and one day by Technologist B.

**Table 5:** Linear equations (slope and intercept) and coefficients of determination (R^2^ values) for each HPV type. Corresponding linearity plots are presented in Figure 1.

HPV Type	Equation	R^2^ Value
HPV16	Y = 0.8868 × X + 2.518	0.9996
HPV18	Y = 0.9843 × X + 22.50	0.9962
HPV31	Y = 1.003 × X − 3.387	0.9987
HPV33	Y = 1.088 × X − 10.92	0.9982
HPV35	Y = 0.9892 × X + 10.03	0.9991
HPV39	Y = 0.9368 × X − 0.05201	0.9991
HPV45	Y = 0.8978 × X + 0.5441	0.9978
HPV51	Y = 0.9955 × X − 2.500	0.9998
HPV52	Y = 0.9918 × X + 0.5093	0.9984
HPV56	Y = 0.9314 × X + 3.252	0.9973
HPV58	Y = 0.9030 × X + 3.403	0.9996
HPV59	Y = 0.9982 × X + 0.09592	0.9986
HPV66	Y = 0.9376 × X − 2.244	0.9968
HPV68	Y = 0.9086 × X − 0.9594	0.9971

**Figure A1:**
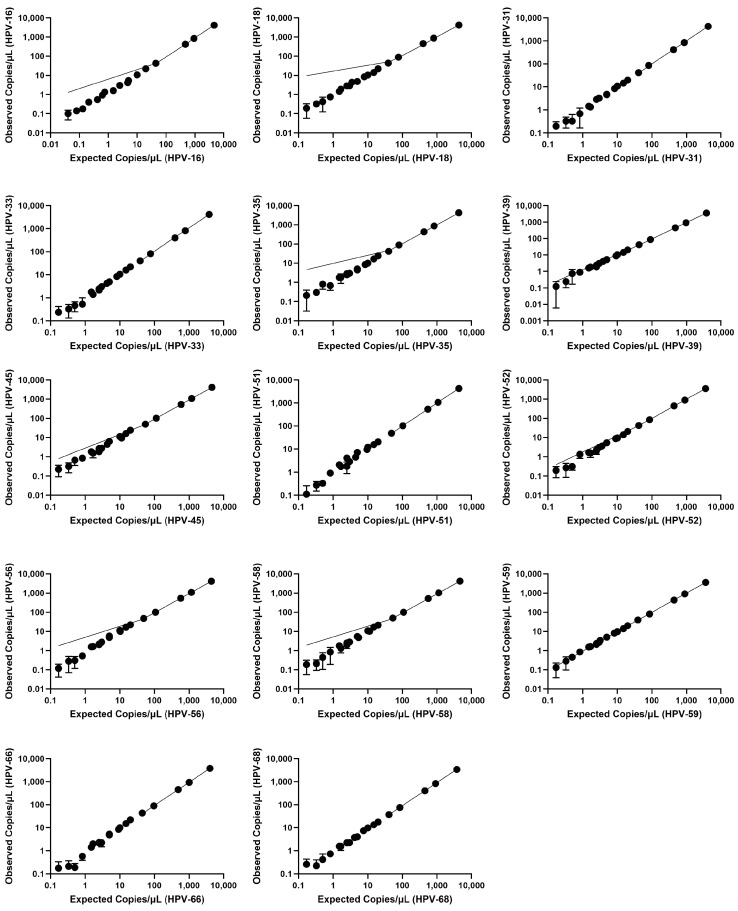
Linearity across the entire detectable concentration range of NavCerDX. The corresponding equations and R^2^ values are provided in Table A2.

## Data Availability

This manuscript and Appendix A and Appendix B include all of the raw data.

## Appendix A. Dilution Series for Analytical Validation Parameters: Accuracy, Precision, Linearity, LoD, and LoQ

**Table A1:** Dilution series of high- and low-concentration samples used to determine accuracy, precision, linearity, limit of detection (LoD), and limit of quantitation (LoQ).

	Dilution Series Used to Determine Accuracy, Precision, and Linearity (copies/µL)													
Concentration	HPV Type													
Level *	16	18	31	33	35	39	45	51	52	56	58	59	66	68
**1**	4696	4317	4240	3822	4277	3790	4611	4299	3582	4431	4616	3605	4026	3747
**2**	940	808	862	775	832	973	1187	1076	895	1160	1122	891	1009	915
**3**	468	400	422	393	423	476	589	546	441	574	561	439	486	449
**4**	-	77	81	77	80	91	113	104	86	107	109	86	96	84
**5**	44	39	41	39	40	43	55	49	42	49	53	40	45	41
**6**	-	7.9	8.4	8.2	8.4	9.2	11.3	9.6	9.0	10.3	11.3	8.3	9.2	7.5
**7**	4.7	3.5	5.0	4.2	5.0	3.9	4.3	4.5	3.6	5.0	5.4	3.3	5.0	4.2
**8**	20	20	20	20	20	20	20	20	20	20	20	20	20	20
**9**	-	15	15	15	15	15	15	15	15	15	15	15	15	15
**10**	10	10	10	10	10	10	10	10	10	10	10	10	10	10
**11**	5	5	5	5	5	5	5	5	5	5	5	5	5	5
**12**	-	3	3	3	3	3	3	3	3	3	3	3	3	3
**13**	2.5	2.5	2.5	2.5	2.5	2.5	2.5	2.5	2.5	2.5	2.5	2.50	2.50	2.50
**14**	-	1.7	1.7	1.7	1.7	1.7	1.7	1.7	1.7	1.7	1.7	1.67	1.67	1.67
**15**	1.5	1.5	1.5	1.5	1.5	1.5	1.5	1.5	1.5	1.5	1.5	1.50	1.50	1.50
**16**	-	0.83	0.83	0.83	0.83	0.83	0.83	0.83	0.83	0.83	0.83	0.83	0.83	0.83
**17**	-	0.50	0.50	0.50	0.50	0.50	0.50	0.50	0.50	0.50	0.50	0.50	0.50	0.50
**18**	-	0.33	0.33	0.33	0.33	0.33	0.33	0.33	0.33	0.33	0.33	0.33	0.33	0.33
**19**	-	0.17	0.17	0.17	0.17	0.17	0.17	0.17	0.17	0.17	0.17	0.17	0.17	0.17
**20**	0.75	-	-	-	-	-	-	-	-	-	-	-	-	-
**21**	0.63	-	-	-	-	-	-	-	-	-	-	-	-	-
**22**	0.42	-	-	-	-	-	-	-	-	-	-	-	-	-
**23**	0.21	-	-	-	-	-	-	-	-	-	-	-	-	-
**24**	0.13	-	-	-	-	-	-	-	-	-	-	-	-	-
**25**	0.08	-	-	-	-	-	-	-	-	-	-	-	-	-
**26**	0.04	-	-	-	-	-	-	-	-	-	-	-	-	-

***** Concentration levels 1 to 7 represent the actual concentrations determined by ddPCR prior to conducting the validation experiments. Concentration levels 8 to 26 represent the expected concentrations derived from the dilution of a validated higher concentration as determined by ddPCR. Certain concentration levels were not prepared for all HPV types.

## Appendix B. Linearity Across the Entire Detectable Concentration Range

To extend the linearity graph to encompass the lowest detectable analyte concentration, data from linearity, limit of detection (LoD), and limit of quantitation (LoQ) experiments were integrated. The initial linearity experiment assessed concentrations ranging from approximately 4000 copies/µL to 4 copies/µL for all human papillomavirus (HPV) types (Appendix A). Subsequently, LoD/LoQ calculations were performed in two phases: the first phase examined five concentrations between ~20 and 1.5 copies/µL (for HPV-16) and six concentrations between 20 and 1.5 copies/µL (for HPV types 18, 31, 33, 35, 39, 45, 51, 52, 56, 58, 59, 66, and 68), while the second phase focused on seven concentrations between approximately ~0.75 and 0.04 copies/µL (for HPV-16) and ~3 and 0.17 copies/µL (for HPV types 18, 31, 33, 35, 39, 45, 51, 52, 56, 58, 59, 66, and 68) (Table A2). Figure A1 illustrates linearity across the entire detectable concentration range. The corresponding equations and R^2^ values are provided in Table A3.

**Table A2:** Dilution series in LoQ/LoD experiment.

Concentration Levels in the First Phase of the LoQ/LoD Experiment	
HPV16	HPV Types 18, 31, 33, 35, 39, 45, 51, 52, 56, 58, 59, 66, and 68
20	20
10	15
5	10
2.5	5
1.5	2.5
	1.5
**Concentration Levels in the Second Phase of the LoQ/LoD Experiment**	
**HPV16**	**HPV Types 18, 31, 33, 35, 39, 45, 51, 52, 56, 58, 59, 66, and 68**
0.75	3.00
0.63	2.50
0.42	1.67
0.21	0.83
0.13	0.50
0.08	0.33
0.04	0.17

**Table A3:** Linear equations (slope and intercept) and coefficients of determination (R^2^ values) for each HPV type. Corresponding linearity plots are presented in Figure A1.

HPV Type	Equation	R^2^ Value
HPV16	Y = 0.8872 × X + 1.262	0.9997
HPV18	Y = 0.9880 × X + 9.602	0.9967
HPV31	Y = 1.002 × X − 1.407	0.9989
HPV33	Y = 1.086 × X − 4.624	0.9984
HPV35	Y = 0.9909 × X + 4.416	0.9992
HPV39	Y = 0.9368 × X + 0.09014	0.9993
HPV45	Y = 0.8977 × X + 0.6558	0.9981
HPV51	Y = 0.9950 × X − 0.8600	0.9999
HPV52	Y = 0.9919 × X + 0.2239	0.9986
HPV56	Y = 0.9318 × X + 1.669	0.9977
HPV58	Y = 0.9035 × X + 1.781	0.9996
HPV59	Y = 0.9983 × X − 0.01611	0.9988
HPV66	Y = 0.9371 × X − 0.8443	0.9973
HPV68	Y = 0.9084 × X − 0.4254	0.9975
